# Integrating soil imaging with spatial omics to uncover root–soil interactions

**DOI:** 10.1016/j.xplc.2026.101890

**Published:** 2026-05-07

**Authors:** Tino Colombi, Alix Vidal, Hannah V. Cooper, Rahul A. Bhosale

**Affiliations:** 1 School of Biosciences, University of Nottingham, LE12 5RD Nottingham, UK; 2 Soil Biology Group, Wageningen University and Research, AA 6700 Wageningen, the Netherlands

**Keywords:** rhizosphere, root–soil interactions, spatial omics, correlative imaging, soil heterogeneity

## Abstract

Soils exhibit remarkable spatial heterogeneity in environmental conditions, which plants perceive at the levels of the whole root system, individual roots, and root tissues. Cropping practices aimed at reducing the environmental footprint of agriculture are likely to intensify this heterogeneity, highlighting the urgent need to adapt crops to heterogeneous soil environments. Recent advances in soil imaging and spatial omics offer unprecedented opportunities to decipher the molecular, physiological, and ecological processes that underpin plant–soil interactions. In this review, we explore the substantial yet largely untapped potential of integrating soil imaging with spatial omics to uncover the fundamental mechanisms that control root foraging in heterogeneous soils. We present an overview of key imaging and molecular approaches that have particular potential for revealing root foraging behavior. To demonstrate their capabilities for generating spatially explicit insights into root–soil interactions, we highlight selected case studies covering both biotic (beneficial and detrimental soil organisms) and abiotic (physical and chemical soil properties) factors. Finally, we outline a workflow for integrating spatial omics with soil imaging through vertical integration of experimental studies across levels of environmental complexity, coupled with predictive modeling. Unlocking the full potential of these approaches will require linking molecular, physiological, and ecological mechanisms at the root–soil interface to whole-plant growth and crop productivity. These fundamental insights into the edaphic drivers of root foraging will be essential for guiding crop adaptation to future, more heterogeneous soil environments.

## Teaser

The integration of soil imaging with spatial omics offers unprecedented, yet largely unexplored, opportunities to elucidate how plants interact with their belowground environment. This review highlights recent developments in soil imaging and spatial omics and outlines a roadmap for multimodal, cross-scale integration of techniques to achieve a more comprehensive understanding of root foraging behavior in complex and heterogeneous soil environments.

## Multiscale heterogeneities define root–soil systems

Most terrestrial plants, including all major crop species, acquire water and nutrients from the soil, making soils an essential resource for local and global agricultural production (Kopittke et al., 2019). Climate change, soil degradation, and resource scarcity, which limit the availability of fertilizers and irrigation water, highlight the urgent need to adapt existing crop germplasm to specific edaphic conditions (Lynch et al., 2022). Root systems that enable efficient acquisition of water and nutrients from the soil are pivotal for improving future food security and agricultural sustainability (Wang et al., 2020). Yet, despite widespread recognition of the crucial importance of root–soil interactions for whole-plant growth and productivity (Mehra et al., 2025), soils remain severely underrepresented in plant breeding research (Raza et al., 2025). Interdisciplinary research bridging plant biology and soil-system science is essential for understanding how to leverage the belowground mechanisms that underpin plant growth to adapt crops to specific soil environments (Wang et al., 2020; Colombi et al., 2024; Dini-Andreote et al., 2025).

The remarkable spatial heterogeneity of soil properties and conditions is a defining feature of the root environment, and it is this spatial heterogeneity that distinguishes the belowground from the aboveground environment of plants (Walter et al., 2009; Mehra et al., 2025). Leaves and stems are exposed to strong diel and seasonal fluctuations in light intensity, temperature, and vapor pressure deficit. Although soils are not static environments, temporal fluctuations in environmental conditions are much less pronounced belowground than aboveground. Yet, soil properties and conditions that regulate root growth and the availability of water and nutrients exhibit substantial spatial variability, even at submillimeter scales (Wang et al., 2020). Spatial soil heterogeneities emerge from the complex interactions among plants, soil microorganisms, and soil fauna (e.g., earthworms, arthropods, and nematodes); soil physical and chemical properties and processes; and local climatic and geological conditions (York et al., 2016; Vetterlein et al., 2020; Mueller et al., 2024). These interactions result in environmental gradients within the soil, the formation of soil pockets with specific environmental conditions, and, ultimately, the development of spatially distinct ecological niches (Ebrahimi and Or, 2016; Borer et al., 2018; Dini-Andreote et al., 2025).

**Figure 1:**
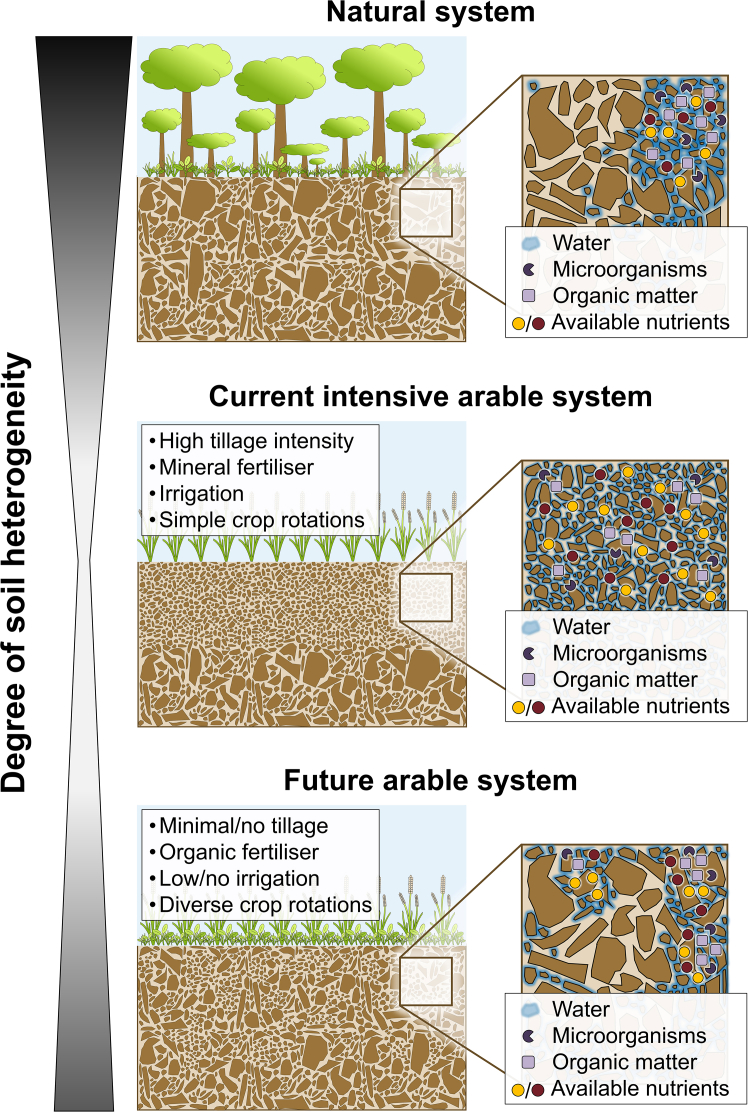
Schematic representation of natural, current intensive, and future arable systems, illustrating the effects of management intensity on the spatial heterogeneity of soil properties and conditions that collectively shape the belowground environment of plants. Sustainable practices in future systems, such as reduced tillage, the (partial) replacement of mineral fertilizers with organic fertilizers, and more diverse crop rotations, are expected to partially restore soil heterogeneity, bringing arable systems closer to conditions found in natural ecosystems.

Tillage, irrigation, fertilization, and reliance on simple crop rotations with low genetic diversity reduce the spatial variability in soil properties and conditions. Therefore, belowground environmental conditions tend to be more homogeneous in intensively managed arable systems than in natural systems, especially in the topsoil (Figure 1). Increasing resource scarcity, as well as environmental regulations and incentives aimed at reducing the environmental footprint of agriculture, will reduce the future use of external inputs such as fuel, fertilizer, and irrigation water. Lower management intensity is accompanied by a reduction in anthropogenic disturbance of soil processes, fostering the development of spatial niches with distinct environmental conditions within soil and thus greater soil heterogeneity (Van Der Bom et al., 2020; Or et al., 2021; Zhang et al., 2022a). Hence, future arable systems are likely to be characterized by greater belowground heterogeneity than current intensively managed systems, more closely resembling natural soil environments (Figure 1).

**Figure 2:**
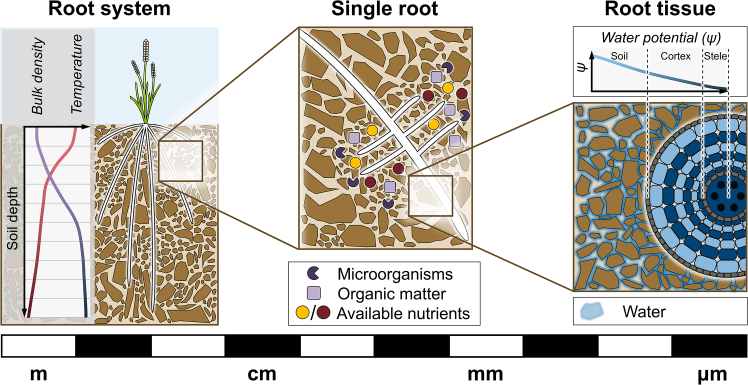
Multiscale heterogeneity, from meters to micrometers, of soil properties and conditions perceived by individual plants at the root-system, single-root, and root-tissue scales. Illustrative examples include (left) soil bulk density and temperature gradients along the soil profile; (middle) localized hotspots of high organic matter concentration, microbial activity, and nutrient availability; and (right) water potential gradients across the rhizosphere and root tissues.

Spatial differences in parent material and local topography cause between- and within-field soil heterogeneities (Ulrich and Becker, 2006; Suriyavirun et al., 2019; Ito et al., 2023). However, we focus here on soil heterogeneities (gradients and spatially distinct pockets) that are relevant at the single-plant level, encompassing spatial variability from the root system scale down to the root-tissue scale (Lynch, 1995; Wang et al., 2020; Colombi et al., 2024). Belowground environmental conditions change with soil depth, leading to distinct environments for shallow and deep roots of the same plant. For example, soil density increases with depth owing to overburden pressure, whereas temperature decreases (left panel in Figure 2). Lower temperature and greater bulk density both reduce root growth rates and thereby limit plant access to water and nutrients in deeper soil layers (Kautz et al., 2013; Lynch and Wojciechowski, 2015).

Localized high concentrations of soil organic matter lead to the emergence of millimeter- to centimeter-sized pockets with greater nutrient availability due to locally increased biological activity and symbiosis between plants and soil organisms such as mycorrhizal fungi (Kuzyakov and Blagodatskaya, 2015; Kuzyakov and Razavi, 2019; Mueller et al., 2024). Owing to these so-called “microbial hotspots,” a single root may grow into and out of spatially distinct zones whose environmental conditions differ substantially from the surrounding soil (center panel in Figure 2). Moreover, plants contribute to the development of these hotspots through locally increased root growth and exudation, leading to synergies between the localized activities of roots and soil organisms (Paterson et al., 2006). The soil directly influenced by living roots, i.e., the rhizosphere, is characterized by environmental gradients at the micro- to millimeter scale (York et al., 2016; Kuzyakov and Razavi, 2019; Vetterlein et al., 2020; Mueller et al., 2024), which often extend into and continue within the different root tissues. For example, water potential within the rhizosphere decreases toward the root surface and then from the outer cortical tissue into the vasculature in the center of the root (right panel in Figure 2; Swaef et al., 2022). Ultimately, these multiscale spatial heterogeneities, which occur as fine gradients and distinct pockets, shape the edaphic basis of crop growth and productivity.

Recent advances in imaging (Tracy et al., 2020; Ahkami et al., 2024) and molecular techniques (Barmukh et al., 2025) have yielded a suite of tools for quantifying the effects of soil heterogeneities on plant ecological, physiological, and molecular processes. For example, nanoscale secondary ion mass spectrometry (NanoSIMS) and micro-X-ray fluorescence spectroscopy (μXRF) provide two-dimensional (2D) insights into the spatial distribution of chemical elements, including plant nutrients, in soil (Mueller et al., 2013; van Veelen et al., 2020). X-ray micro-computed tomography (μCT) enables non-destructive quantification of the spatial arrangement of different soil constituents, including mineral and organic particles, living roots, and the soil pore system (Phalempin et al., 2025). Imaging can also be used for spatially explicit assessments of root physiological processes, referred to as “spatial phenomics,” including the interactions of roots with the soil pore space via X-ray μCT (Colombi et al., 2017; Lucas et al., 2023) and root water uptake patterns through neutron imaging (Cai et al., 2022). Spatial transcriptomics, proteomics, and metabolomics provide insights into molecular processes and mechanisms across different root classes and tissues, revealing mechanisms that underpin the interactions of roots with their environment (Marcon et al., 2015; Li et al., 2021; Wang et al., 2024; Zhu et al., 2025). Moreover, spatially explicit metagenomic approaches allow researchers to link the bulk soil and rhizosphere microbiomes with the endophytic microbiomes of different root tissues (Wang et al., 2024). The combination of spatial omics techniques with cutting-edge soil imaging tools provides new capabilities for identifying the key belowground mechanisms that determine water and nutrient capture and whole-plant growth in heterogeneous soil environments.

Despite their enormous potential for deciphering root–soil interactions and their effects on crop productivity, spatial omics and advanced soil imaging approaches are rarely, if ever, combined. In this review, we highlight the unique possibilities offered by integrating spatial omics with cutting-edge imaging techniques to reveal the key belowground mechanisms governing plant growth. To this end, we first provide an overview of recent developments and applications of soil imaging and spatial omics approaches used to quantify root–soil interactions from the root-system scale to the root-tissue scale. We then present selected case studies that demonstrate the ability of these approaches to generate novel insights into root foraging behavior and whole-plant growth in heterogeneous soil environments. Finally, we outline strategies to facilitate the integration of spatial omics and soil imaging to provide the fundamental insights needed to adapt crops to future, more heterogeneous soil environments, thereby improving the sustainability and resilience of cropping systems.

## Imaging techniques to visualize and quantify soil heterogeneity

Several comprehensive reviews of the use of imaging to visualize and quantify spatial soil heterogeneities across scales have been published in recent years, and readers are referred to these reviews for detailed information (Vetterlein et al., 2020; Li et al., 2022a; Ahkami et al., 2024). Below, we provide a brief overview of selected techniques that show particular promise for deciphering the implications of spatial soil heterogeneity for root foraging behavior and whole-plant growth (Table 1).

**Table 1:** Overview of different soil imaging techniques, their resolution, and the insights they offer for resolving root–soil interactions.

Technique	2D/3D	Spatial resolution	Fields of application	Key limitations	Example studies
Nanoscale secondary ion mass spectrometry (NanoSIMS)	2D	50 nm	tracing isotopes in root–microorganism–soil systems; mapping the spatial distribution of elements in a complex organo-mineral matrix	requires dehydrated and vacuum-stable samples; captures a small sample area (often <0.04 mm^2^)	Kaiser et al. (2015); Vidal et al. (2018); Hestrin et al. (2019)
Micro-X-ray fluorescence spectroscopy (μXRF)	2D	5–25 μm	spatial distribution of metals and nutrients in root–soil systems	limited sensitivity for light elements such as C and N	Castillo-Michel et al. (2012); van Veelen et al. (2020); Lippold et al. (2023)
Planar optodes	2D	100 μm	real-time, non-invasive imaging of chemical gradients (e.g., O_2_, pH, CO_2_); root–soil interface in transparent rhizoboxes at cm level	membrane attached to soil surface leading to boundary layer	Blossfeld et al. (2011); Bereswill et al. (2024)
Zymography	2D	100 μm	visualizing enzyme activity *in situ* at the mm to cm scale; semi-quantitative functional mapping using color intensity	limited to specific enzymes and substrates; membrane attached to the soil surface leading to a boundary layer	Spohn and Kuzyakov (2013); Ghaderi et al. (2025); Lattacher et al. (2025)
Neutron radiography/tomography	2D/3D	50–200 μm (resolution decreases with greater sample size)	two- or three-dimensional neutron attenuation maps; non-invasive quantification of water fluxes in soil–root systems	mineral soil matrix not clearly detectable; relatively low spatial resolution; more complex than X-ray-based systems	Zarebanadkouki et al. (2013),(2014); Tötzke et al. (2017)
Synchrotron radiation/X-ray micro-computed tomography (SRCT/μCT)	3D	1–200 μm (resolution decreases with greater sample size)	three-dimensional X-ray attenuation maps reflecting material density; non-invasive quantification of major soil constituents	water and organic material cannot be distinguished on the basis of X-ray attenuation signals; X-ray radiation affects biological processes	Koebernick et al. (2017); Schlüter et al. (2022); Phalempin et al. (2025)
Positron emission tomography	3D	>1000 μm (resolution decreases with greater sample size)	three-dimensional mapping of positron-emitting isotopes, e.g., ^11^C, for non-invasive quantification of carbon fluxes along the shoot–root–soil axis	low spatial resolution; soil matrix and pores are not directly detectable; very limited access to^11^C	Jahnke et al. (2009); Yin et al. (2020)

### Visualizing chemical elements and soil (bio-)chemical gradients

NanoSIMS enables ultra-high-resolution visualization (down to 50 nm) of multiple isotopes of different chemical elements in soil (Mueller et al., 2013). This enables the tracing of carbon fluxes from roots to soil (Vidal et al., 2018; Shabtai et al., 2024) and nutrient uptake from the soil by microorganisms and plants (Clode et al., 2009; Hestrin et al., 2019). However, owing to its limited surface coverage, generalizing and upscaling the findings obtained with NanoSIMS remain challenging. Furthermore, sample preparation, whether through, for example, resin embedding or deposition of soil samples onto wafers, is labor intensive and inevitably alters aspects of the sample’s structure and chemical composition. The destructive nature of these procedures also precludes *in situ* analyses on the same specimen (Mueller et al., 2013; Höschen et al., 2015; Védère et al., 2022; Li et al., 2023). μXRF enables mapping of the spatial distribution of various metals and nutrients at the root–soil interface over larger areas than NanoSIMS at a resolution of 5–25 μm (<10 μm for synchrotron-based systems). However, detection of lighter elements such as carbon and nitrogen with μXRF is difficult (van Veelen et al., 2020; Schlüter et al., 2022).

In contrast to static techniques such as NanoSIMS and μXRF, planar optodes provide non-invasive insights into the temporal dynamics of chemical gradients across soil surfaces spanning several square centimeters. These sensors can visualize and quantify gradients in pH, O_2,_ or CO_2_ at approximately 100 μm resolution (Blossfeld et al., 2011; Zhu et al., 2014; Bereswill et al., 2024). At comparable spatial resolutions and scales, zymography enables *in situ* visualization of enzyme activities (e.g., phosphatases and proteases) at the root–soil interface (Spohn and Kuzyakov, 2013; Razavi et al., 2019). Together, planar optodes and zymography offer unique, spatially explicit insights into the biogeochemical processes that control soil nutrient cycling (Bilyera et al., 2022). 2D soil drying and plant water uptake patterns can be visualized using neutron radiography at resolutions of 100–150 μm, using heavy water (D_2_O) as a tracer (Zarebanadkouki et al., 2013, 2014; Ahmed et al., 2018).

### Resolving the 3D configuration of soil constituents and processes

X-ray μCT and, to a lesser extent, synchrotron radiation X-ray computed tomography (SRCT) have been widely adopted in soil science. Both techniques can simultaneously visualize and co-locate key soil constituents such as plant roots, organic residues, the mineral soil matrix, and the soil pore space (Schlüter et al., 2022; Phalempin et al., 2025). Depending on the sample size, resolutions down to 1–10 μm are achievable, enabling researchers to quantify the impact of root growth and root hair formation on rhizosphere microstructure development and dynamics (Aravena and Berli, 2010; Keyes et al., 2017; Koebernick et al., 2017). Moreover, μCT and SRCT enable the study of temporal changes in the soil pore system due to moisture fluctuations (Pires et al., 2020), root growth, and organic matter decomposition (Koebernick et al., 2017; Lucas et al., 2023). It is important to note that, while non-destructive, μCT and SRCT are not non-invasive, as X-ray radiation affects both plants and soil organisms. Consideration of these effects is particularly important when investigating the temporal dynamics of biological processes through repeated scanning, and lead shielding is advised to minimize X-ray exposure to the field of view (Zappala et al., 2013; Ganther et al., 2020; Lippold et al., 2021).

X-ray μCT has also been used for spatially explicit quantification of water fluxes in soil (Tracy et al., 2015; Schwenk et al., 2023). However, because the signal in μCT and SRCT scans is determined mainly by material density, distinguishing water from roots and other organic residues is difficult, if not impossible. Hence, the suitability of μCT and SRCT for studying root–soil water relationships is limited. Neutron tomography uses D_2_O as a tracer to track 3D water flow in soil and water uptake by plants at resolutions of approximately 100 μm (Moradi et al., 2011; Tötzke et al., 2017). Positron emission tomography uses the positron-emitting carbon isotope ^11^C to quantify carbon fluxes from the shoot to the root and into the surrounding soil in three dimensions (Jahnke et al., 2009; Yin et al., 2020). However, owing to the relatively coarse resolution (>1000 μm) and the highly specialized equipment needed to produce ^11^C, positron emission tomography is used far less frequently than other soil imaging approaches.

### Correlative imaging and integration with mathematical modeling

Given the strengths and limitations of individual soil imaging techniques, combining different techniques on the same sample, i.e., “correlative imaging,” is particularly promising for capturing the complex interactions that shape soil environments (Oburger and Schmidt, 2016). For example, integration of μCT or SRCT with chemical imaging techniques such as NanoSIMS and μXRF enables researchers to elucidate linkages between the spatial configuration of soil constituents (e.g., roots, organic residues, and pores) and the emergence of millimeter- to micrometer-scale biochemical gradients in soil (Schlüter et al., 2019; Keyes et al., 2022; Lippold et al., 2023). Moreover, the combination of correlative imaging with mathematical modeling using mechanistic and empirical approaches enables the prediction of how soil heterogeneities affect water and nutrient transport and plant uptake of these resources (Koebernick et al., 2017; Duddek et al., 2023; Lippold et al., 2025). For more detailed information on image-informed modeling, we refer readers to Ahkami et al. (2024), who provide a comprehensive overview of different approaches and recent developments.

## Spatial omics for functional root and rhizosphere profiling

As with soil imaging techniques, the application of omics approaches in root and rhizosphere research has been comprehensively reviewed in recent years (Tracy et al., 2020; Li et al., 2022b; Kimotho and Maina, 2024; Sena et al., 2024; Nobori, 2025; Srikanth et al., 2025; Yusuf et al., 2025). Therefore, in this section, we focus mainly on selected techniques that show particular potential for providing spatially explicit insights into interactions at the root–soil interface that govern root function and foraging behavior (Table 2).

**Table 2:** Overview of different omics techniques that provide spatially resolved insights into root responses to edaphic cues and stresses.

Technique	Fields of application	Limitations	Key references
Spatial image-assisted root phenomics (e.g., X-ray computed tomography, neutron imaging, microscopy techniques)	information on the spatial configuration of physiological and/or structural root traits	sample throughput is relatively low, and data processing and analysis are time intensive; limited applicability for large-scale (screening) studies	Ahmed et al. (2018); Strock et al. (2019); Fusi et al. (2022); Lucas et al. (2023); Duddek et al. (2023)
Spatial transcriptomics	mapping gene expression across root zones and tissues; identifying molecular responses to environmental cues	high cost and technical complexity; limited resolution of some platforms; requires specialized sample preparation	Yu et al. (2016); Ganther et al. (2022); Zhong et al. (2025)
Spatial proteomics	profiling protein abundance and localization in root tissues and cell types; linking gene expression to functional outcomes	sample preparation is challenging; single-cell resolution still emerging; proteome coverage may be incomplete	Marcon et al. (2015); Montes et al. (2024)
Spatial metabolomics	mapping metabolite distribution across root tissues; understanding biochemical and stress-adaptation pathways	metabolite identification can be ambiguous; spatial resolution varies by technique; dynamic metabolite changes could complicate interpretation	Sarabia et al. (2018); Oyarce et al. (2025); Yang et al. (2025)
Spatial metagenomics and metatranscriptomics	profiling microbial diversity and activity across soil compartments, root classes, and root tissuesunderstanding root–microbiome interactions	limited resolution at the single-cell level; challenges in linking microbial identity to functionrequires complex sampling and bioinformatics	Muscatt et al. (2022); Wang et al. (2024); Galindo-Castañeda et al. (2025)

### Belowground spatial plant phenomics

Imaging has become an essential tool for plant science in recent years, especially for the extraction of spatially explicit information on plant physiological processes (Walter et al., 2015; Tardieu et al., 2017; Tracy et al., 2020; Li et al., 2022b), an approach we refer to as “spatial phenomics.” Belowground spatial phenomics uses imaging techniques similar to those used in soil science to study root traits and physiological processes *in situ*. For example, X-ray μCT enables non-destructive visualization of root architecture and the soil pore system, revealing how spatial features of the pore system influence root growth patterns and overall foraging behavior (Colombi et al., 2017; Atkinson et al., 2020; Lucas et al., 2023). At finer scales, SRCT combined with image-based modeling has been used to study the role of root hairs in water uptake during soil drying (Duddek et al., 2023). As discussed above, the effects of X-ray exposure on molecular and physiological processes in plants must be considered, especially when plants are repeatedly exposed to X-rays (Zappala et al., 2013; Ganther et al., 2020). Neutron radiography enables the quantification of water uptake by different root classes, offering insights into functional differentiation within the root system (Ahmed et al., 2018). Moreover, standard bright-field microscopy and laser ablation tomography are powerful tools for assessing root-class-specific anatomical responses to edaphic cues and stresses such as drought, soil compaction, and colonization by soil organisms (Strock et al., 2019; Vanhees et al., 2020; Koehler et al., 2025). These microscopy techniques also provide high-resolution data on tissue-specific traits that are critical for understanding root responses and acclimation to edaphic cues and stresses.

Importantly, the above techniques enable the study of root–soil interactions while preserving the natural spatial context of the root–soil interface, which cannot be captured in homogeneous gel- or agar-based systems. Although soil-based imaging approaches present greater technical and analytical challenges due to soil opacity, physical heterogeneity, and strong resource gradients, the insights gained under realistic growth conditions outweigh these difficulties. Soil-based spatial phenomics can reveal spatially patterned root traits, localized stress responses, and potentially emergent root–soil–microbe interactions that remain inaccessible in simplified experimental systems. By maintaining the natural spatial organization of the root–soil interface, these approaches enable the identification of functionally relevant, cell- and tissue-specific acclimation processes that underpin root foraging behavior and stress resilience in agricultural soils.

### Spatially resolved molecular processes in the plant root system

Molecular spatial omics techniques, including transcriptomics, proteomics, and metabolomics, have emerged as powerful tools for dissecting the molecular complexity of root systems in response to developmental and environmental cues. These approaches enable tissue- and cell-specific profiling of gene expression, protein abundance, and metabolite distribution, providing fine-scale insights into root functions and responses to edaphic cues.

Spatial transcriptomics maps gene expression across root zones and tissues, revealing specific molecular responses to biotic and abiotic stresses. For example, laser capture microdissection integrated with RNA sequencing of pericycle cells from primary, seminal, crown, and brace roots showed root-type-specific transcriptomic reprogramming in response to local high nitrate; this was associated with different lateral root branching patterns, with brace roots showing the strongest architectural plasticity (Yu et al., 2016). Similarly, Ganther et al. (2022) demonstrated that maize root epidermal and root-hair-forming cells adjust their gene expression in response to soil texture and sampling depth. Using a soil column platform with two textures (loam and sand), three depths, and wild-type plants versus the root-hair-defective *rth3* mutant, the authors showed that texture was the strongest driver of root transcriptomes, followed by depth, whereas the effect of root hairs was reflected in genes associated with epidermal cell differentiation, cell wall organization, and defense responses. Together, these results highlight local, soil-context-dependent changes in maize gene expression that shape nutrient and water acquisition and modulate root–soil interactions. More recently, comparative spatial transcriptomics of upland and irrigated rice using Stereo-seq revealed ecotype-specific programs in the coleoptile node and root tip that underpin crown-root formation and development. In particular, the transcriptional regulator HMGB1 emerged as a key factor, promoting root elongation and thickening and thereby enhancing drought resistance (Zhong et al., 2025).

Spatial proteomics connects gene expression to protein function by profiling protein abundance and localization within root tissues. For example, a high-resolution, tissue-specific proteome and phosphoproteome atlas of maize primary roots identified functional gradients along the longitudinal axis (meristematic and elongation zones) and tissue specialization across the cortex and stele. These data delineate zones of protein and phosphoprotein abundance relevant to processes such as cell-wall formation, transport, stress responses, and secondary metabolism, with cortex-specific enrichment of proteins involved in flavonoid biosynthesis (Marcon et al., 2015). In *Arabidopsis*, single-cell proteomics resolved cortex versus endodermal identities by quantifying approximately 3200 proteins at single-cell resolution and identifying ∼600 proteins enriched in either cell type, thereby distinguishing closely related root cell types and revealing tissue-specific protein networks (Montes et al., 2024).

Spatial metabolomics complements these approaches by mapping metabolites across tissues and cell types, offering insights into biochemical activities and stress adaptation. For example, matrix-assisted laser desorption/ionization mass spectrometry imaging of barley seminal roots subjected to high salinity (150 mM NaCl) resolved longitudinal, zone-specific metabolic reprogramming. This included marked shifts in phosphatidylcholine species (e.g., a reduction in PC 34:n) and simultaneous ionic changes (increased Na^+ and^ decreased K^+^ content), illustrating how spatially resolved chemistry underpins salt responses in developing roots (Sarabia et al., 2018). Yang et al. (2025) integrated single-cell transcriptomics with spatial metabolomics in *Panax* root tips, reconstructing early cell-type differentiation (including endodermal trajectories), revealing epidermis-specific expression of key ginsenoside biosynthetic enzymes, and functionally implicating *IAA29* in endodermis suberization and *MYB2/MYB78* in ginsenoside biosynthesis. In the same study, mass spectrometry imaging mapped the spatial accumulation of ginsenosides across root-tip cell types. Oyarce et al. (2025) integrated microscopy with spatial metabolomics in *Avicennia marina* and *Phoenix dactylifera* roots, revealing tissue- and species-specific metabolic strategies under salinity and drought. These included suberin and lignin accumulation in mangrove meristems, enrichment of amino acid biosynthesis in date palm, and localization of osmoprotectants to the exodermis and epidermis in both mangrove and date palm roots.

Overall, these spatial omics studies reveal how defined cell types and tissues execute distinct molecular and metabolic programs that underpin root acclimation to soil heterogeneity, nutrient stress, and environmental challenges. Nevertheless, current spatial omics platforms operate at resolutions that may not fully resolve all functionally relevant cell-type-specific responses.

### Spatial configuration of the root and rhizosphere microbiome

The rhizosphere is a hotspot for interactions between plants and microorganisms. Advances in spatial metagenomics and metatranscriptomics now enable high-resolution mapping of microbial diversity and activity across soil compartments, root classes, and tissues, providing new insights into the spatial organization of microbial functions. For example, 16S rRNA sequencing in conjunction with root transcriptomic profiling in maize demonstrated that microbial communities assemble in a compartment-specific manner from bulk soil to the rhizosphere to root tissues (Wang et al., 2024). Similarly, the maize root microbiome was shown to change along individual root axes, highlighting the effects of localized exudation patterns and soil conditions on the emergence of distinct microbial communities (Galindo-Castañeda et al., 2025). Zhang et al. (2025) provided evidence that spatially distinct root exudation patterns lead to functional zonation of microbial communities, thereby affecting localized phosphorus solubilization.

Spatial meta-omics has also been used to study viral communities within the rhizosphere. Muscatt et al. (2022) combined viromics, metagenomics, and metatranscriptomics to reveal the spatial and temporal dynamics of DNA and RNA viruses across bulk soil, rhizosphere, and root compartments. Their findings revealed that viral activity increases near crop roots and may modulate bacterial metabolism, introducing the concept of viral priming in response to crop rotation. Together with metagenomics and metatranscriptomics studies, which are revolutionizing our understanding of microbial interaction networks and chemical diversity in the rhizosphere (Boparai and Sharma, 2021), recent *in situ* soil zymography studies (e.g. Lattacher et al., 2025) have shown how contrasting root architectures, such as deep versus shallow root systems, influence microbial biomass and enzyme activities in the topsoil and subsoil. Collectively, these studies reinforce the importance of spatial resolution in root–microbe interaction research.

## Emerging insights from soil imaging techniques and spatial omics

We selected five case studies that highlight the potential of advanced soil imaging, spatial omics, and their combinations to fundamentally advance our understanding of the interactions between roots and their heterogeneous environment. These examples include both abiotic and biotic edaphic cues and span the different scales at which soil heterogeneities are perceived by plants (i.e. the root-system, single-root, and root-tissue scales; Figure 2). The first three studies demonstrate the capabilities of soil imaging or spatial omics when used in isolation, and the fourth and fifth illustrate how soil imaging approaches can be combined with different omics techniques.

### Example 1: *In situ* visualization of the emergence of chemical gradients around plant roots

Combining a suite of chemical imaging techniques with X-ray μCT scanning, Lippold et al. (2023) visualized carbon and nutrient gradients in the rhizosphere. Maize (*Zea mays*) was grown in soil columns labeled with ^15N-^enriched nitrate and pulse-labeled with ^13^CO_2_. X-ray μCT scanning at low (45 μm) and high (10 μm) resolutions guided the selection of sampling locations for chemical imaging and enabled quantification of the physical structure of the rhizosphere. Using NanoSIMS to trace ^13^C and ^15^N, the authors showed that plant-derived carbon was released up to 100 μm into the rhizosphere, whereas soil nitrogen primarily accumulated in root cells. Moreover, μXRF revealed gradients of decreasing calcium and sulfur concentrations from the root surface to the surrounding soil, suggestive of gypsum (CaSO_4_·2H_2_O) precipitation at the root surface. The innovative combination of 2D and 3D imaging demonstrated the enormous potential of correlative imaging to provide new *in situ* insights into the processes that determine rhizosphere functioning. By integrating these approaches, the study showed that carbon release and nutrient redistribution closely align with microscale physical features of the rhizosphere—mechanistic insights that cannot be obtained using any single imaging technique alone. However, a limited field of view, low throughput, and destructive sampling are inherent limitations of this correlative workflow, highlighting the need for complementary developments that enable insights across root types and developmental stages.

### Example 2: Root-tissue-specific colonization by edaphic organisms

Strock et al. (2019) demonstrate the potential of laser ablation tomography to quantify the colonization of roots and specific tissues by beneficial and detrimental soil organisms. These included arbuscular mycorrhizal (AM) fungi, the western corn rootworm (*Diabrotica virgifera virgifera*), the cereal cyst nematode (*Heterodera avenae*), and *Fusarium virguliforme* in roots of maize, barley, and common bean, respectively. The system used a 355-nm UV laser source to generate 3D images of root anatomical structures at 1–5 μm resolution. Laser excitation induced tissue-specific autofluorescence, enabling differentiation between colonizing organisms and affected or unaffected root tissues. This capacity enabled precise localization of arbuscules and nematodes in the cortex, as well as the identification of tissue-specific damage caused by rootworm, nematode, and *Fusarium* infection. By including a range of edaphic organisms, Strock et al. (2019) demonstrated the unique capabilities of laser ablation tomography to resolve tissue-scale interactions between roots and their biotic environment, revealing how specific microbes and pathogens occupy distinct tissue domains. Such anatomical information could be combined with spatial transcriptomics or proteomic datasets in future integrative studies to link colonization patterns to tissue-specific molecular responses. Because laser ablation tomography is destructive and provides only endpoint snapshots, it cannot capture colonization dynamics, highlighting the need for complementary non-invasive imaging approaches to enable fully integrated temporal and spatial analyses.

### Example 3: Spatially resolved gene expression during root–fungus symbiosis

Serrano et al. (2024) investigated the molecular dynamics of AM symbiosis between *Medicago truncatula* and *Rhizophagus irregularis*. Plants were grown and inoculated with AM fungal spores in a sand–clay mixture that was amended with nutrient solution. Using integrated single-nucleus RNA sequencing and spatial transcriptomics, they generated spatially registered maps of infected and uninfected root cell types. Their study revealed that cortex cells exhibit distinct transcriptomic profiles during different stages of fungal colonization, highlighting the dynamic interplay between host and symbiont. Spatial clusters within the root tissue showed differential expression of genes responsive to AM fungi, including phosphate transporters and fungal markers, enabling the identification of early and late stages of symbiosis. This study demonstrates the power of spatial transcriptomics to resolve multi-kingdom interactions at cellular resolution and provides a valuable resource for understanding nutrient exchange and stress resilience in root–fungus symbioses—insights that would be difficult to capture with bulk transcriptomics alone. However, because spatial transcriptomics provides only static snapshots, complementary time-resolved approaches are needed to fully capture the temporal progression of these interactions.

### Example 4: Profound effects of root–soil contact on soil carbon inputs

Lucas et al. (2023) investigated the effects of root–soil pore interactions on rhizodeposition in switchgrass (*Panicum virgatum*) and yellow coneflower (*Rudbeckia hirta*) grown in pots filled with sieved and naturally structured soil. To quantify the architecture of the soil (macro-)pore system, root foraging behavior, and rhizodeposition, they combined high-resolution X-ray μCT (5–18 μm) with ^14^CO_2_ plant labeling and subsequent ^14^C tracing. Their results showed that greater root growth into the soil matrix (rather than into existing macropores) and the concomitant increase in root–soil contact stimulate rhizodeposition and soil microbial activity. These findings provided mechanistic evidence that the co-localization of plant roots and soil pore networks mediates carbon allocation patterns in plant–soil systems that underpin terrestrial carbon and nutrient cycling. By combining detailed analysis of the soil pore system with spatial phenomics, Lucas et al. (2023) set a benchmark for future studies on the linkages between soil heterogeneity and root foraging behavior—insights that neither imaging nor isotope tracing alone could capture. However, capturing longer-term changes in pore dynamics remains challenging, highlighting the need for scalable, reproducible approaches to generalize these findings across species, soil types, and environmental conditions.

### Example 5: Divergent selection affects hydropatterning intensity in maize roots

Hydropatterning is a key acclimative response of roots to heterogeneous water availability, involving the preferential initiation of lateral roots toward moist surfaces. Using a germination-paper-based assay, Scharwies et al. (2025) observed substantial differences in the intensity of hydropatterning in a maize (*Zea mays*) diversity panel (>200 inbred lines) that covered most of the genetic diversity in current public maize breeding programs. They then deployed X-ray μCT scanning for *in situ* validation of these findings in a subset of inbred lines with contrasting hydropatterning intensities. To do so, they quantified hydropatterning in roots that grew inside soil macropores and were partially in contact with the moist pore wall and air. Population structure analysis using whole-genome sequencing data revealed that hydropatterning intensity was greater in tropical and subtropical lines than in temperate lines. Moreover, data from field trials showed that stronger hydropatterning could increase rooting depth. This integration of soil and root imaging with genetic information across various scales showed that plant breeding can promote preferential root growth toward locally available water. Because this study links a water-responsive developmental mechanism to natural genetic variation and field-relevant phenotypes, it exemplifies the type of cross-scale, integrative research encouraged in this review. However, μCT-based validation remains labor intensive and difficult to scale to large breeding populations, highlighting the need for more rapid imaging frameworks or trait-linked markers to enable high-throughput selection during crop improvement.

## An integrative workflow for moving from mechanisms to holistic pattern detection

**Figure 3:**
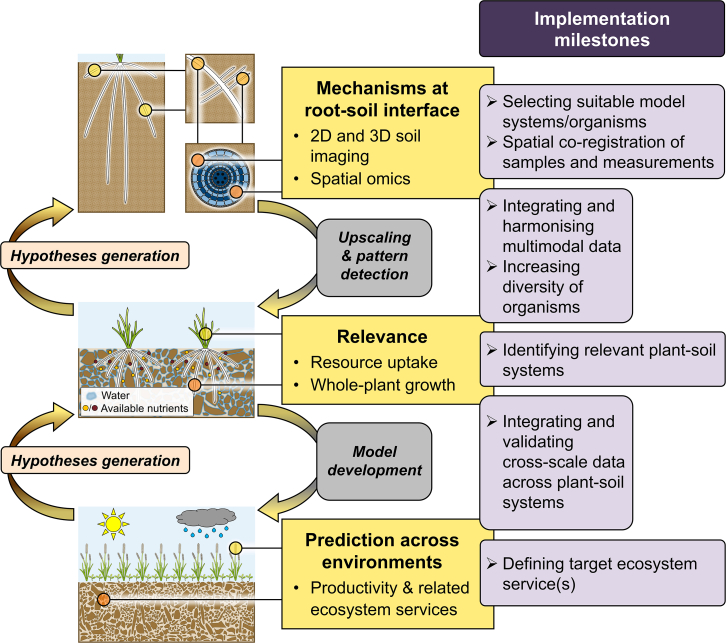
Illustration of the proposed workflow and key implementation milestones for vertically integrating experimental studies across increasing levels of environmental complexity with model development. Mechanisms at the root–soil interface are identified by combining spatial omics and soil imaging in experiments performed under controlled conditions using soil analogs or simple soil-filled columns. These findings are then upscaled to the whole-plant and/or plot level to test the relevance of the identified mechanisms and detect patterns that link localized plant molecular, physiological, and ecological processes with surrounding soil properties and conditions. Experimental data obtained across levels of environmental complexity inform the development of mathematical models to predict plant performance and related ecosystem services across diverse arable environments. Cross-scale integration is further catalyzed through downstream interactions, i.e., the generation of hypotheses based on *in silico* predictions or observations made at the field or mesocosm scale.

Root foraging behavior emerges from the complex interactions between plants and the soil environment and from the effects of these interactions on plant molecular, physiological, and ecological processes (Dini-Andreote et al., 2025; Mehra et al., 2025). To reveal the implications of root–soil interactions for water and nutrient acquisition, we must account for the spatial heterogeneity of soil environments at the root-tissue, single-root, and root-system scales. An integrative workflow that covers molecular, physiological, and ecological processes from the tissue to the root-system scale will therefore be indispensable for linking mechanisms at the root–soil interface to whole-plant growth and crop productivity (Figure 3).

### The transformative potential of integrating spatial omics and soil imaging

Efficient soil exploration by roots is essential for crop adaptation to greater spatial soil heterogeneity, which is likely to be a key feature of future arable systems. The heterogeneous distribution of plant-available nutrients and water in future, less intensively managed arable soils (Figure 1) will require co-localization of root growth, exudation, and mycorrhizal symbiosis with available soil resources to ensure efficient soil exploration (Wang et al., 2020; Zhang et al., 2022a; Colombi et al., 2024). Spatially explicit assessment of the processes that underpin root–soil interactions, such as the co-localization of lateral root placement or mycorrhizal symbiosis with available resources, is undoubtedly an ambitious endeavor. Nonetheless, interdisciplinary research that combines recent advances in spatial omics and soil imaging offers unparalleled opportunities to address this challenge. The integration of different cutting-edge imaging approaches with molecular techniques holds enormous potential to reveal the fundamental molecular, physiological, and ecological mechanisms that shape root–soil interactions in heterogeneous environments. Below, we propose vertical integration of experimental studies across increasing levels of environmental complexity, together with model development that can facilitate this integration (Figure 3).

Experiments under controlled environmental conditions are essential for pinpointing the specific mechanisms that control root foraging behavior (e.g., Bhosale et al., 2018, Dietrich et al., 2017, Giri et al., 2018, Xiong et al., 2025). Mimicking soil heterogeneities found in the field with soil analogs or simple soil-filled columns permits the identification of genes and molecular signaling pathways that enable plants to sense and respond to localized edaphic cues (e.g., Kiers et al., 2011; Orosa-Puente et al., 2018; Stanley et al., 2018; Zhang et al., 2022b; Mehra et al., 2022; Scharwies et al., 2025). Upscaling findings obtained under controlled environments to the mesocosm and field-plot scales allows us to verify and quantify the relevance of specific mechanisms for soil exploration, resource acquisition, and whole-plant growth (McKenzie et al., 2009; Colombi et al., 2017; Schneider et al., 2021; Siegwart et al., 2025; van der Bom et al., 2025). The empirical insights obtained across levels of environmental complexity can then guide the development and parameterization of predictive mathematical models to extrapolate findings across a wide range of environmental conditions (Schnepf et al., 2022; Ahkami et al., 2024). Informing model development with spatial omics and soil imaging data will unlock transformative insights into the molecular, physiological, and ecological mechanisms at the root–soil interface that shape root foraging behavior. Furthermore, downstream interactions, that is, designing experiments based on *in silico* predictions or observations made at the field or mesocosm scale, will catalyze novel insights into the functioning of plant–soil systems (Figure 3).

The successful implementation of the proposed integrative workflow will require careful consideration of several key points. These include the selection of suitable model systems and organisms to identify mechanisms at the root–soil interface that shape root foraging behavior, as well as the identification of relevant plant–soil systems to verify the relevance of these mechanisms for whole-plant growth and productivity. Similarly, target ecosystem services will need to be clearly defined when developing computational tools to predict the role and importance of root–soil interactions for the functioning of plant–soil systems. Moreover, the integration, harmonization, and validation of multimodal data, i.e., data obtained using different image modalities and omics approaches across relevant spatial and temporal scales, will be essential for the upscaling and extrapolation of findings from simple to increasingly complex environments (Figure 3).

### Key challenges for the successful integration of spatial omics and soil imaging

Although the transformative potential of integrating spatial omics with soil imaging is evident, several key challenges hamper their adoption. Imaging and, to a lesser extent, molecular techniques are often time and resource intensive. Imaging approaches such as NanoSIMS, μXRF, SRCT, and X-ray μCT require careful and time-consuming sample preparation and image acquisition, which limit the achievable sample throughput (Schlüter et al., 2019; Vetterlein et al., 2021; Védère et al., 2022). Similarly, the quantification of spatial soil heterogeneity from images can be time consuming (Védère et al., 2022; Ahkami et al., 2024). Because of these time requirements and the generally high purchase and operating costs of imaging equipment, imaging is costly, and this limits its widespread applicability. However, the rapid development of artificial intelligence tools and their adoption in soil imaging and root phenomics are greatly expediting the automation and standardization of image analysis (Steffens et al., 2017; Han et al., 2021; Phalempin et al., 2025), fostering the use of imaging techniques. Furthermore, imaging techniques such as planar optodes and zymography provide insights into the spatial configuration of soil environments at much lower cost and potentially higher throughput than NanoSIMS, μXRF, SRCT, or X-ray μCT (Blossfeld et al., 2011; Spohn and Kuzyakov, 2013; Bereswill et al., 2024; Ghaderi et al., 2025; Lattacher et al., 2025). The processing and analysis of molecular data are much more standardized than those of imaging data, allowing much higher sample throughput. Moreover, the current costs associated with molecular techniques such as spatial transcriptomics, proteomics, and metabolomics, which limit their large-scale applicability, are expected to decrease in the coming years, thereby facilitating the use of molecular techniques (Song et al., 2023).

Soil imaging and omics approaches typically generate large and often highly complex multivariate datasets. First, data acquired using different image modalities and molecular techniques must be harmonized to enable the detection of patterns that connect image-derived and molecular information (Atkinson et al., 2019; Vetterlein et al., 2020; Alexandrov et al., 2023). Similar challenges exist for the integration of different modeling approaches, such as gene regulatory network models and process-based soil models that simulate belowground environmental conditions and plant–soil interactions. Moreover, pattern recognition itself is challenging and often requires considerable computing power owing to the sheer volume and the complexity of the data. Progress in artificial intelligence and increasing computing power will be key facilitators for detecting the spatial patterns that link localized plant molecular, physiological, and ecological processes with surrounding soil properties and conditions (Pound et al., 2017; Yan and Wang, 2023). However, to make predictions about the effects of different root foraging behaviors on whole-plant growth and productivity across diverse environments, we must move beyond purely correlative, data-mining-driven approaches (Roose et al., 2016). Hence, rigorous mechanistic frameworks, obtained from and tested in controlled experiments, must provide the basis for data-driven pattern recognition and model development (Figure 3).

In addition to these technical challenges, differences in research traditions and cultures between soil and plant science can hamper the integration of spatial omics with soil imaging. For example, reductionist approaches are widespread in plant science, especially among molecular plant biologists. Soil science, on the other hand, tends to embrace a more systems-based perspective (Vetterlein et al., 2020). Efforts to establish common ground between disciplines and to make plant scientists an integral part of the global change research community, such as “PlantACT” (Hirt et al., 2023), are essential for facilitating the integration of spatial omics with the soil imaging techniques we advocate here.

## Concluding remarks and future perspectives

The environmental conditions in which plants forage for water and nutrients are defined by the spatial heterogeneities in soil properties and conditions and the resulting interactions at the root–soil interface (Vetterlein et al., 2020; Mueller et al., 2024). These heterogeneities are perceived by individual plants at the root-tissue, single-root, and root-system levels and collectively affect molecular, physiological, and ecological processes in the plant–soil system (Wang et al., 2020; Colombi et al., 2024; Dini-Andreote et al., 2025). Unlike aboveground environmental conditions, which are very similar across an agricultural field, belowground environmental conditions vary greatly between individual plants in the same field. Therefore, the responses and interactions of individual plants with their complex spatial environment must guide efforts to improve soil exploration and water and nutrient uptake in crops.

A holistic understanding of root foraging behavior requires identification of the key mechanisms that drive complex interactions at the root–soil interface and upscaling of these mechanisms to the whole-plant and field scales. Key research questions in this endeavor include the following.

• What are the specific molecular signals and pathways that govern root acclimation to localized water and nutrient deficiencies and their localized interactions with soil biota in heterogeneous soil environments?
• (How) can we develop “live” imaging approaches that capture molecular and physiological processes in the rhizosphere in real time under conditions that resemble or mimic natural soil environments?
• How do plant–microbe interactions specifically shape the physical and chemical properties of the rhizosphere, and how these feedback loops influence localized carbon and nutrient cycling?
• How do spatial gradients in root exudate composition along individual roots shape rhizosphere microbial communities, and how do spatial gradients in microbial community composition feed back on spatial soil heterogeneity?
• Can we move beyond microbial communities and incorporate larger soil fauna, such as earthworms, soil-dwelling arthropods, and nematodes, into our understanding of the ecological interactions that shape rhizosphere properties and functions?
• How do root–soil interactions at the tissue, single-root, and root-system scales translate into whole-plant performance and field-scale ecosystem services?
• (How) can we leverage advanced computational tools such as artificial intelligence approaches to build and parameterize predictive models of root growth and function in spatially complex soil environments, thereby moving beyond correlative observations?
• What specific molecular signals and regulatory networks mediate root responses to localized soil heterogeneities, and how can spatial omics be combined with imaging-derived information on microenvironments to disentangle these processes?
• How can gene regulatory network models, hormone-signaling dynamics, and microbial functional networks be integrated with pore-scale and root-system-scale imaging to produce predictive, cross-scale models of root foraging?
• How can spatial omics, imaging, and modeling be merged into a practical, cross-scale framework that enables upscaling from microenvironments to whole-plant and field-scale predictions?
• How can integrative approaches help disentangle the combined roles of microbes, soil fauna, and root structural and physiological plasticity in shaping rhizosphere structure and function?

We fully acknowledge that the spatial complexity of soil environments can be overwhelming and that the idea of adapting crops to such complex environments may seem far-fetched. However, we now have a suite of methods at our disposal that enable us to spatially resolve and quantify soil environmental conditions and their interactions with molecular, physiological, and ecological processes in roots. Combining cutting-edge soil imaging with spatial omics techniques across scales opens numerous new possibilities for better understanding root foraging behavior in heterogeneous soil environments and its implications for whole-plant growth and crop productivity. Ultimately, integrating cutting-edge soil imaging with spatial omics across scales will unlock a mechanistic understanding of root foraging in heterogeneous soils, informing breeding programs aimed at adapting crops to future soil environments.

## Funding

T.C. acknowledges financial support from the University of Nottingham (Nottingham Research Fellowship). R.B. acknowledges support from a BBSRC Discovery Fellowship (BB/S011102/1) and BBSRC New Investigator Research (BB/X014843/1) and partner (BB/X018806/1) grants. H.C. acknowledges support from BBSRC Growing Health (BBS/E/RH/230003B) and BBSRC Delivering Sustainable Wheat (BBS/E/RH/230001A). R.B., T.C., and H.C. acknowledge a UK-CG project funded by UK International Development from the UK government and BBSRC, part of UK Research and Innovation.

## Acknowledgments

The views expressed do not necessarily reflect the UK government’s official policies. No conflict of interest declared.

## Author contributions

R.A.B. initiated the review. T.C. and R.A.B. conceived the idea for the review. The overall structure was developed by T.C. and R.A.B. with input from A.V. and H.V.C. T.C. and R.A.B. wrote the initial draft. All authors contributed to writing the original draft and to reviewing and editing the manuscript.
